# Can the Responses of Photosynthesis and Stomatal Conductance to Water and Nitrogen Stress Combinations Be Modeled Using a Single Set of Parameters?

**DOI:** 10.3389/fpls.2017.00328

**Published:** 2017-03-28

**Authors:** Ningyi Zhang, Gang Li, Shanxiang Yu, Dongsheng An, Qian Sun, Weihong Luo, Xinyou Yin

**Affiliations:** ^1^College of Agriculture, Nanjing Agricultural UniversityNanjing, China; ^2^Center for Crop Systems Analysis, Department of Plant Sciences, Wageningen University & ResearchWageningen, Netherlands; ^3^Horticulture and Product Physiology Group, Department of Plant Sciences, Wageningen University & Research CentreWageningen, Netherlands

**Keywords:** mesophyll conductance, model, nitrogen, photosynthesis, stomatal conductance, water

## Abstract

Accurately predicting photosynthesis in response to water and nitrogen stress is the first step toward predicting crop growth, yield and many quality traits under fluctuating environmental conditions. While mechanistic models are capable of predicting photosynthesis under fluctuating environmental conditions, simplifying the parameterization procedure is important toward a wide range of model applications. In this study, the biochemical photosynthesis model of Farquhar, von Caemmerer and Berry (the FvCB model) and the stomatal conductance model of Ball, Woodrow and Berry which was revised by Leuning and Yin (the BWB-Leuning-Yin model) were parameterized for *Lilium* (*L. auratum* × *speciosum* “Sorbonne”) grown under different water and nitrogen conditions. Linear relationships were found between biochemical parameters of the FvCB model and leaf nitrogen content per unit leaf area (*N*_a_), and between mesophyll conductance and *N*_a_ under different water and nitrogen conditions. By incorporating these *N*_a_-dependent linear relationships, the FvCB model was able to predict the net photosynthetic rate (*A*_n_) in response to all water and nitrogen conditions. In contrast, stomatal conductance (*g*_s_) can be accurately predicted if parameters in the BWB-Leuning-Yin model were adjusted specifically to water conditions; otherwise *g*_s_ was underestimated by 9% under well-watered conditions and was overestimated by 13% under water-deficit conditions. However, the 13% overestimation of *g*_s_ under water-deficit conditions led to only 9% overestimation of *A*_n_ by the coupled FvCB and BWB-Leuning-Yin model whereas the 9% underestimation of *g*_s_ under well-watered conditions affected little the prediction of *A*_n_. Our results indicate that to accurately predict *A*_n_ and *g*_s_ under different water and nitrogen conditions, only a few parameters in the BWB-Leuning-Yin model need to be adjusted according to water conditions whereas all other parameters are either conservative or can be adjusted according to their linear relationships with *N*_a_. Our study exemplifies a simplified procedure of parameterizing the coupled FvCB and *g*_s_ model that is widely used for various modeling purposes.

## Introduction

In the past decades, many crop models have been developed for predicting yield in response to changing environments. Some studies evaluated the performance of different crop models under different growth conditions such as different temperature, water supply and soil fertility (Jamieson et al., 1998; Adam et al., 2011; Palosuo et al., 2011). Surprisingly, when testing these models under a large land scale or long time span, the yield predictions in most models turned out to be an artifact of the balance between incorrect predictions of assimilation and leaf area index (Jamieson et al., 1998) or between biomass production and harvest index (Palosuo et al., 2011). The radiation-use efficiency approach that was taken in many crop models may over-simplify underlying processes and a more detailed approach, based on quantitative functional relationships for underlying processes, is needed in order to capture the effects of high temperature and high radiation intensities on crop growth under changing environments (Challinor et al., 2009; Adam et al., 2011). While detailed models usually require more effort in terms of model parameterization, some parameters and functional relationships are found to change very little (i.e., are conservative) among crop types (von Caemmerer et al., 2009) and environmental conditions (Yin, 2013). Therefore, it is important to test the conservative level of commonly used functional relationships, so as to balance between the level of detail in these models and the efforts needed for model parameterization.

Photosynthesis is the primary physiological process that drives crop growth and productivity and influences many plant quality traits, and is strongly affected by environmental factors. Accurately predicting photosynthesis is the first step toward predicting crop growth, yield and quality in response to environmental changes. Water and nitrogen variations frequently occur in crop fields. The effects of water and nitrogen on photosynthesis have been extensively and separately studied (Grassi et al., 2002; Xu and Baldocchi, 2003; Gu et al., 2012). The combined effect of water and nitrogen on photosynthesis, however, has received less attention.

Previous modeling studies have shown that the use of empirical factors to capture the effect of stresses, does not model photosynthesis reliably in many cases (Jamieson et al., 1998). The effects of environmental factors on leaf photosynthesis can be best investigated by use of the biochemical model of Farquhar, von Caemmerer and Berry (the FvCB model hereafter) (Farquhar et al., 1980) combined with diffusion models. The FvCB model has been widely used to describe photosynthesis in response to multiple environmental changes (Harley et al., 1992; Grassi et al., 2002; Xu and Baldocchi, 2003; Monti, 2006; Qian et al., 2012). The model describes photosynthesis as the minimum of the Rubisco-limited rate and the electron transport-limited rate. Major parameters in this model are the maximum Rubisco carboxylation rate (*V*_cmax_, definitions of all model variables hereafter are listed in Table 1), the maximum electron transport rate (*J*_max_) and the mitochondrial day respiration (*R*_d_). These biochemical parameters have been found to be linearly correlated with leaf nitrogen content per unit leaf area (*N*_a_) under environmental changes such as various nitrogen supply (Grassi et al., 2002; Yin et al., 2009) and elevated CO_2_ (Harley et al., 1992; Yin, 2013), as well as seasonal changes (Zhu et al., 2011). However, whether or not the linear relationships between these biochemical parameters and *N*_a_ exist under drought is debatable, mainly due to inconsistent effect of drought on *N*_a_ (Díaz-Espejo et al., 2006; Damour et al., 2008, 2009).

**Table 1:** **List of model variables and their definitions and units**.

**Variable**	**Definition**	**Unit**
*A*_c_	Rubisco carboxylation-limited net photosynthetic rate	μmol CO_2_ m^−2^ s^−1^
*A*_j_	Electron transport-limited net photosynthetic rate	μmol CO_2_ m^−2^ s^−1^
*A*_n_	Net photosynthetic rate	μmol CO_2_ m^−2^ s^−1^
*a*_1_	Ratio of *C*_i_ to *C*_a_ for vapor saturated air	–
*b*_1_	Decreasing slope of *C*_i_/*C*_a_ ratio with the increase of VPD	kPa^−1^
*C*_a_	Ambient CO_2_ level	μbar
*C*_c_	CO_2_ level in the chloroplast	μbar
*C*_i_	Intercellular CO_2_ level	μbar
*C*_i*_	*C*_i_-based CO_2_ compensation point in the absence of *R*_d_	μbar
*D*_Jmax_	Deactivation energy of *J*_max_	J mol^−1^
*D*_gm_	Deactivation energy of *g*_m_	J mol^−1^
*E*_gm_	Activation energy of *g*_m_	J mol^−1^
*E*_Jmax_	Activation energy of *J*_max_	J mol^−1^
*E*_KmC_	Activation energy of *K*_mC_	J mol^−1^
*E*_KmO_	Activation energy of *K*_mO_	J mol^−1^
*E*_Rd_	Activation energy of *R*_d_	J mol^−1^
*E*_Vcmax_	Activation energy of *V*_cmax_	J mol^−1^
*f*_cyc_	Fraction of electrons at PSI following the cyclic transport around PSI	–
*f*_pseudo_	Fraction of electrons at PSI following the pseudocyclic transport	–
$F_{\text{m}}^{\prime }$	Maximum fluorescence	–
*F*_s_	Steady-state fluorescence	–
*g*_m_	Mesophyll conductance	mol m^−2^ s^−1^ bar^−1^
*g*_m25_	Value of *g*_m_ when leaf temperature is 25°C	mol m^−2^ s^−1^ bar^−1^
*g*_s_	Stomatal conductance for CO_2_ diffusion	mol m^−2^ s^−1^
*g*_0_	Residual stomatal conductance when the irradiance approaches to zero	mol m^−2^ s^−1^
*I*_inc_	Incident irradiance	μmol photon m^−2^ s^−1^
*J*	PSII electron transport rate that is used for CO_2_ fixation and photorespiration	μmol e^−^ m^−2^ s^−1^
*J*_max_	Maximum value of *J* under saturating irradiance	μmol e^−^ m^−2^ s^−1^
*J*_max25_	Value of *J*_max_ when leaf temperature is 25°C	μmol e^−^ m^−2^ s^−1^
*K*_mC_	Michaelis-Menten coefficients of Rubisco for CO_2_	μbar
*K*_mC25_	Value of *K*_mC_ when leaf temperature is 25°C	μbar
*K*_mO_	Michaelis-Menten coefficients of Rubisco for O_2_	mbar
*K*_mO25_	Value of *K*_mO_ when leaf temperature is 25°C	mbar
LMA	Leaf mass per area	g m^−2^
*N*_a_	Leaf nitrogen content per unit leaf area	g N m^−2^ leaf
*N*_b_	Base leaf nitrogen content at or below which *A*_*n*_ is zero	g N m^−2^ leaf
*O*	Partial pressures of O_2_ in the chloroplast	mbar
*R*	Universal gas constant (= 8.314)	J K^−1^ mol^−1^
*R*_d_	Mitochondrial day respiration	μmol CO_2_ m^−2^ s^−1^
*R*_d25_	Value of *R*_*d*_ when leaf temperature is 25°C	μmol CO_2_ m^−2^ s^−1^
*s*	Factor used to calculate electron transport rate from chlorophyll fluorescence	–
*S*_Jmax_	Entropy term of *J*_max_	J K^−1^ mol^−1^
*S*_gm_	Entropy term of *g*_m_	J K^−1^ mol^−1^
*T*	Leaf temperature	°C
*V*_cmax_	Maximum Rubisco carboxylation rate	μmol CO_2_ m^−2^ s^−1^
*V*_cmax25_	Value of *V*_cmax_ when leaf temperature is 25°C	μmol CO_2_ m^−2^ s^−1^
VPD	Vapor pressure deficit	kPa
χ_*J*_	Slope of the linear relationship between *J*_max25_ and *N*_a_	μmol e^−^ (g N)^−1^ s^−1^
χ_*V*_	Slope of the linear relationship between *V*_cmax25_ and *N*_a_	μmol CO_2_ (g N)^−1^ s^−1^
ϕ_**2**_	Apparent operating efficiency of PSII photochemistry	mol e^−^ (mol photon)^−1^
Γ_*_	CO_2_ compensation point in the absence of *R*_d_	μbar
κ_2LL_	Conversion efficiency of incident light into *J* at strictly limiting light	mol e^−^ (mol photon)^−1^
θ	Convexity factor for response of *J* to *I*_inc_	–
β	Absorptance of light by leaf photosynthetic pigments	–
ρ_2_	Proportion of absorbed light partitioned to PSII	–

*PSI, photosystem I; PSII, photosystem II*.

The FvCB model itself requires the CO_2_ concentration in the chloroplast (*C*_c_) as an input variable. To this end, estimating stomatal conductance (*g*_s_) and mesophyll conductance (*g*_m_) is necessary to enable the FvCB model to predict photosynthesis using the atmospheric CO_2_ level (*C*_a_) as input. The stomatal conductance model of Ball et al. (1987) (the BWB-type model hereafter), as one of the most commonly used models of *g*_s_, is often coupled with the FvCB model (Harley et al., 1992; Kosugi et al., 2003). In the BWB-type model, *g*_s_ responds to net photosynthetic rate, relative humidity and CO_2_ concentration at the leaf surface. Although it is phenomenological, the BWB-type model is widely used to model *g*_s_ at leaf level (e.g., Leuning, 1995) and is the most feasible yet biologically robust tool for extrapolating *g*_s_ at the field or forest stand level (Misson et al., 2002; Alton et al., 2007). The original BWB-type model does not capture stomatal responses to soil water status, thus some efforts were made toward modifying the BWB-type model to predict *g*_s_ under drought. Either the slope used in the BWB-type model (describing the response of *g*_s_ to photosynthetic rate, relative humidity or vapor pressure deficit (VPD) and CO_2_ concentration) (Tuzet et al., 2003; Maseyk et al., 2008; Héroult et al., 2013) or the residual stomatal conductance (the value of *g*_s_ when irradiance approaches to zero) (Misson et al., 2004) was reported to decrease under drought, and was related to soil moisture or leaf water potential (Baldocchi, 1997; Wang and Leuning, 1998; Misson et al., 2004; Keenan et al., 2010; Egea et al., 2011; Li et al., 2012; Zhou et al., 2013; Müller et al., 2014). In another study, however, neither of these two parameters was affected by drought (Xu and Baldocchi, 2003). So far, there is no consensus as to how to adjust the BWB-type model parameters to properly model *g*_s_ under drought. Moreover, there are very few studies that investigated the responses of these parameters to nitrogen supply and to the combination of water and nitrogen supply.

*g*_m_ has been considered as infinite in most early studies, in which intercellular CO_2_ concentration (*C*_i_) was used to substitute *C*_c_ in the FvCB model (Harley et al., 1992; Kosugi et al., 2003). However, this assumption has later been proved not true since *C*_c_ is lower than *C*_i_ (Warren, 2004). Ignoring *g*_m_ leads to the underestimation of *V*_cmax_, especially under stress conditions such as drought (Monti, 2006). *g*_m_ has been found to decrease under water-deficit conditions and low nitrogen availability in many previous studies (reviewed in Flexas et al., 2008). There have been only a few attempts to incorporate the effect of drought on *g*_m_ in the photosynthesis model by using a dependence of *g*_m_ on *g*_s_ (Cai et al., 2008) based on the observation of a close correlation between *g*_s_ and *g*_m_ in response to water-deficit conditions (Flexas et al., 2002; Warren, 2008; Perez-Martin et al., 2009) or by including an empirical soil moisture dependent function for *g*_m_ (Keenan et al., 2010). Given that so far no consensus exists, more investigations are needed to incorporate the responses of *g*_m_ to water and nitrogen variations into the photosynthesis model.

When applying the combined FvCB, *g*_s_ and *g*_m_ model for predicting photosynthetic responses to fluctuating environmental variables, inevitably many parameters need to be quantified. Information about which parameters are conservative and which are variable depending on the treatment is extremely useful for predicting photosynthesis under diverse environmental conditions. Given the previous experience that the FvCB model parameters, once expressed as a function of *N*_a_, are not altered by environmental variables such as elevated [CO_2_] (Yin, 2013), we are particularly interested in examining whether the responses of FvCB, *g*_s_ and *g*_m_ model parameters to water and nitrogen stress can be modeled using a single set of parameters when they are related to leaf nitrogen content. The objectives of this study are (i) to test whether or not water and nitrogen stress combinations change the linear relationships between photosynthetic biochemical parameters and leaf nitrogen content, and (ii) to investigate the responses of stomatal conductance model parameters and mesophyll conductance to different water and nitrogen conditions and to quantify these responses for the purpose of model simplicity. To this end, we used *Lilium* (*L. auratum* × *speciosum* “Sorbonne”) as the test plant, as this plant is commonly grown under low-investment greenhouses where plants are frequently subject to different water and nitrogen regimes.

## Materials and methods

### Plant materials and experimental design

Four experiments with the same type of water and nitrogen treatments were conducted in different growth seasons in a plastic greenhouse located at Nanjing, China (32°N, 118°E) during 2009 to 2011 (Table 2). The greenhouse, covered by anti-drop polyvinyl chloride film, was composed of two spans and east-west oriented with a length of 28 m, span width of 8 m, gutter height of 3 m and arch height of 5 m. Heating pipes were installed during winter season. During summer season, the greenhouse was cooled through natural ventilation and an inner shading screen installed at the position with a distance of 1.0-1.4 m to the top. Temperature, VPD and photosynthetically active radiation are shown in the Supplementary Data (Figures S1–S3). No CO_2_ enrichment was applied, and standard cultivation practices for disease and pest control were used as is common for commercial *Lilium* production in China. *Lilium* bulbs, with a circumference of 14-16 cm, were planted in plastic pots filled with substrates of sand, turf and soil (3:1:1). The physicochemical properties of the substrate are shown in Table 2. The pots, with a depth of 14 cm, upper diameter of 18 cm and bottom diameter of 12 cm, were put on seedling beds (*l* × *w* × *h* = 25.0 m × 1.7 m × 1.0 m) and arranged at a density of 36 plants m^−2^.

**Table 2:** **Detailed information of experimental treatment conditions, physicochemical properties of the growth substrate and measurements**.

	**Exp. 1^a^**	**Exp. 2^b^**	**Exp. 3^b^**	**Exp. 4^b^**
**EXPERIMENTAL TREATMENT CONDITIONS**				
Planting date (dd-mm-yyyy)	27-09-2009	29-11-2009	09-09-2010	05-12-2010
Date of starting water treatment (dd-mm-yyyy)	20-10-2009	25-12-2009	20-10-2010	15-02-2011
Date of starting nitrogen treatment (dd-mm-yyyy)	18-10-2009	12-01-2010	21-10-2010	09-02-2011
Harvesting date (dd-mm-yyyy)	22-01-2010	25-04-2010	02-01-2011	02-05-2011
**PHYSICOCHEMICAL PROPERTIES OF THE GROWTH SUBSTRATE**				
Total N (%)	0.03	0.03	0.02	0.02
Organic C (%)	2.08	2.08	2.24	2.24
Available N (mg kg^−1^)	10.10	10.10	9.67	9.67
Available P (mg kg^−1^)	15.75	15.75	11.42	11.42
Available K (mg kg^−1^)	36.97	36.97	40.38	40.38
Bulk density (g cm^−3^)	1.08	1.08	1.12	1.12
EC (mS cm^−1^)	0.18	0.18	0.20	0.20
pH	6.22	6.22	6.01	6.01

a *In Exp. 1, light response curve, CO_2_ response curve and chlorophyll fluorescence were measured, and the combined measurement of photosynthesis and chlorophyll fluorescence under non-photorespiratory conditions was conducted*.

b *In Exps. 2–4, light response curve and chlorophyll fluorescence were measured*.

Two water levels were used: well-watered conditions, with a soil water potential (SWP) of −4 to −15 kPa according to Li et al. (2012), and water-deficit conditions, with a SWP of −20 to −40 kPa. The SWP at 0.1 m below the soil surface was monitored using tensiometers (SWP-100, Institute of Soil Science, Chinese Academy of Sciences) with three replicates per water level. When the SWP reached its designed lower limit value, plants were irrigated until it reached the designed upper limit value. The SWP at 0.1 m below the soil surface and the corresponding gravimetric soil water content were measured to establish calibration curves. These curves were then used to determine the amount of water required for irrigation. The dates of starting water treatment in the four experiments are shown in Table 2.

At each water level, there were four levels of nitrogen supply: 25, 45, 65, and 85 mg available nitrogen per kg substrate (hereafter N25, N45, N65, and N85, respectively). Nitrogen was added in the substrate as urea taking into account that urea can be converted into nitrate within 1 or 2 days (Harper, 1984). The amount of urea needed was calculated based on the targeted treatment level and the amount of available nitrogen in the substrate (Table 2), and urea was directly spread in the substrate, with the dates shown in Table 2. According to Sun (2013), 65 mg available nitrogen per kg substrate is the optimal level of nitrogen supply in commercial *Lilium* production for the cultivar used in this study. Treatments, with a plot area of 2.0 × 1.5 m^2^ and three replicates per treatment, were arranged in a split-plot design with water level assigned to the main plots and nitrogen level to the sub-plots.

### Gas exchange and chlorophyll fluorescence measurements

Gas exchange was measured on newly fully expanded leaf (the 4th leaf counting from the top downward) at flower bud visible stage using the LI-6400 Portable Photosynthesis System (Li-Cor BioScience, Lincoln, NE, USA) under 21% O_2_. In Experiment 1, both light response curves and *C*_i_ response curves were measured in order to identify any differences in photosynthesis parameter estimation by using these two types of curves. For light response curves, incident irradiance (*I*_inc_) in the leaf cuvette was decreased in the series of 1,500, 1,200, 1,000, 600, 400, 200, 100, 50, 20, and 0 μmol m^−2^ s^−1^, while keeping *C*_a_ at 370 μmol mol^−1^. For *C*_i_ response curves, *C*_a_ was increased stepwise: 50, 100, 150, 200, 250, 380, 650, 1,000, and 1,500, while keeping *I*_inc_ at 800 μmol m^−2^ s^−1^. The microclimate conditions in the leaf chamber were automatically controlled. The CO_2_ concentration and water vapor between leaf and the reference chamber were automatically matched before data were recorded. We found that photosynthesis parameters estimated from *A*_n_-*I*_inc_ curves and *A*_n_-*C*_i_ curves were similar (see Results). Therefore, in Experiments 2–4, only *A*_n_-*I*_inc_ curves were measured, as measurement of *A*_n_-*C*_i_ curves inevitably involves the problem of CO_2_ leakage into and out of the leaf cuvette, which would require additional measurements to correct for.

Chlorophyll fluorescence was simultaneously measured using FMS2 (Hansatech Instruments Ltd, UK) at a similar position on the leaf where gas exchange was measured. The steady-state fluorescence (*F*_s_) was measured under natural radiation level (ranged from 0 to 1,200 μmol m^−2^ s^−1^) and saturating *I*_inc_ (at 1,500 μmol m^−2^ s^−1^) after 3–5 min light adaptation, followed by applying a light pulse > 7,000 μmol m^−2^ s^−1^ for <1 s to measure maximum fluorescence $F_{m}^{\prime }$. The apparent operating efficiency of photosystem II photochemistry (Φ_2_) was calculated as $\Phi_{2}=1-F_{s}/F_{m}^{\prime }$ (Genty et al., 1989).

Due to inadequate environmental control in the low-investment greenhouse, air temperature and VPD hardly stayed constant although they were kept within the range suitable for *Lilium* growth (Figures S1, S2). Therefore, all gas exchange and chlorophyll fluorescence measurements in the four experiments were subjected to variations of temperature and VPD.

In order to convert chlorophyll fluorescence data on Φ_2_ into electron transport rate, combined measurement of gas exchange and chlorophyll fluorescence was conducted using the LI-6400XT Portable Photosynthesis System (Li-Cor BioScience, Lincoln, NE, USA) at low oxygen using a gas blend of 2% O_2_ and 98% N_2_ in the leaf chamber at flower bud visible stage (Experiment 1). *A*_n_−*I*_inc_ curves were measured while keeping *C*_a_ at 1,000 μmol mol^−1^, to create non-photorespiratory conditions. *A*_n_ at high *C*_a_ levels (i.e., 650, 1,000, and 1,500 μmol mol^−1^) at 2% O_2_ was also measured while keeping *I*_inc_ at 800 μmol m^−2^ s^−1^. Φ_2_ was assessed using the same procedure as described above. In order to establish the correlation of estimating *R*_d_ using different methods, combined measurement of gas exchange and chlorophyll fluorescence for *A*_n_−*I*_inc_ curves (under 21% O_2_, keeping *C*_a_ at 370 μmol mol^−1^) was also conducted in Experiment 1. All gas exchange data wherever the set-point *C*_a_ differed from the ambient CO_2_ level were corrected for CO_2_ leakage from measurements using thermally killed leaves.

### Leaf characteristics

After gas exchange and chlorophyll fluorescence measurements, the leaves were cut, and leaf area was measured before being put in the oven at 105°C for 30 min and subsequently at 80°C until constant weight. Leaf nitrogen concentration (for organic nitrogen) was measured by using the Kjeldahl digestion method (Sun, 2013). Briefly, leaf dry samples were ground, and a 0.5 g of ground sample was digested with 30% hydrogen peroxide and 5 mL of concentrated sulphuric acid at 340°C. 10 mL of 10 mol L^−1^ sodium hydroxide was then added for distilling the digested solution. The distillate was titrated using 0.02 mol L^−1^ sulfuric acid, and bromocresol green-methyl red was used as the indicator. Leaf nitrogen content per unit leaf area (*N*_a_, g m^−2)^ was calculated based on leaf nitrogen concentration, leaf dry weight and leaf area.

### Estimation of photosynthetic model parameters

The FvCB model (Farquhar et al., 1980) predicts net photosynthetic rate (*A*_n_) as the minimum of the Rubisco carboxylation-limited rate (*A*_c_) and the electron transport-limited rate (*A*_j_):

(1) $$\begin{array}{r} A_{n}=\min\left(A_{c},\;A_{j}\right) \end{array}$$

(2) $$\begin{array}{r} A_{c}=\frac{\left(C_{c}-\Gamma_{*}\right)V_{cmax}}{C_{c}+K_{mC}\left(1+O/K_{mO}\right)}-R_{d} \end{array}$$

(3) $$\begin{array}{r} A_{j}=\frac{\left(C_{c}-\Gamma_{*}\right)J}{4C_{c}+8\Gamma_{*}}-R_{d} \end{array}$$

where *C*_c_ and *O* are the chloroplast partial pressures of CO_2_ and O_2_, respectively; *K*_mC_ and *K*_mO_ are the Michaelis-Menten coefficients of Rubisco for CO_2_ and O_2_, respectively; *R*_d_ is day respiration; Γ_*_ is the CO_2_ compensation point in the absence of *R*_d_ and was calculated as $0.5O\frac{K_{mC}}{K_{mO}}\left[\text{exp}\left(-3.3801+\frac{5,220}{298R\left(T+273\right)}\right)\right]$ (Yin et al., 2004), derived from the parameter values of Bernacchi et al. (2001); *J* is the photosystem II electron transport rate that is used for CO_2_ fixation and photorespiration.

*R*_d_ was firstly estimated as the *y*-axis intercepts of the linear regression plots of *A*_n_ against *I*_inc_ (the Kok method hereafter) (Sharp et al., 1984). The Kok method tends to underestimate *R*_d_ (Sharp et al., 1984; Yin et al., 2011). Therefore, *R*_d_ was also estimated from the linear regression of *A*_n_ against (*I*_inc_Φ_2_/4) (the Yin method hereafter) (Yin et al., 2009, 2011) using data available from the combined measurement of gas exchange and chlorophyll fluorescence, in order to establish the calibration relationship between values of *R*_d_ estimated by the two methods. As combined gas exchange and chlorophyll fluorescence data were used only in part of our measurements, all the *R*_d_ estimated based on the Kok method was then corrected according to the established calibration relationship to obtain *R*_d_ estimates for all treatments.

The calculation of *A*_c_ or *A*_j_ in the FvCB model requires *C*_c_, which is unknown beforehand. Therefore, *A*_j_ relevant parameters were estimated based on Yin et al. (2009) using chlorophyll fluorescence data. To convert fluorescence-based data on Φ_2_ into electron transport rate *J*, a calibration needs to be made for each water and nitrogen treatment. This was done by linear regression plot of *A*_j_ against (*I*_inc_Φ_2_/4), using data obtained under non-photorespiratory conditions from low light levels of the *A*_n_−*I*_inc_ curve and three high CO_2_ levels. The slope *s* of this linear regression was used as a calibration factor to calculate values of electron transport rate under all conditions: *J* = *sI*_inc_Φ_2_ (Yin et al., 2009). The obtained *J* was then fitted to the following equation to obtain electron transport parameters of the FvCB model:

(4) $$\begin{array}{r} J=\frac{\kappa_{2\text{LL}}I_{\text{inc}}+\;J_{\text{max}}-\sqrt{{\left(\kappa_{2\text{LL}}I_{\text{inc}}+\;J_{\text{max}}\right)}^{2}-4\theta \;J_{\text{max}}\kappa_{2\text{LL}}I_{\text{inc}}}}{2\theta \;} \end{array}$$

where κ_2LL_ is the conversion efficiency of incident light into *J* at strictly limiting light; *J*_max_ is the asymptotic maximum value of *J* when *I*_inc_ approaches to saturating level; θ is a convexity factor for response of *J* to *I*_inc_, and was assumed to have a constant value of 0.8 (Yin and Struik, 2015). Since chlorophyll fluorescence measurement was conducted under fluctuating temperature, the value of *J*_max_ at 25 °C (*J*_max25_) and κ_2LL_ were calculated by combining Equation (4) with Equation (7) (see later) that describes the temperature response of *J*_max_.

With *J*_max25_ and κ_2LL_ calculated as described above, *J*_max_ for each *A*_n_−*I*_inc_ curve from gas exchange measurement was derived according to the temperature level during each measurement using Equation (7) (see later). *J* at each light level in the *A*_n_−*I*_inc_ curve was then derived using Equation (4) based on *J*_max_ and κ_2LL_ calculated before.

With *J* and *R*_d_ calculated, *g*_m_ was then estimated assuming that *g*_m_ was constant across the entire light response curve. Whether or not *g*_m_ is constant across light or CO_2_ levels remains debatable, but this assumption allows the identification of any differences among water and nitrogen treatments in the actual average *g*_m_. For that purpose, a relatively less measurement error-sensitive method, the NRH-A method (Yin and Struik, 2009a), was used to estimate the value of *g*_m_ as constant, by fitting the following non-rectangular hyperbolic (NRH) equation for the *A*_j_ part of the *C*_i_-based FvCB model:

(5) $$\begin{array}{l} A=0.5\{x_{1}-R_{d}+g_{m}(C_{i}+x_{2})\\ \;\;\;\;\;\;-\sqrt{\begin{array}{l} \;\;\;\;\;{\left[x_{1}-R_{d}+g_{m}(C_{i}+x_{2})\right]}^{2}-\\ 4g_{m}[(C_{i}-\Gamma_{*})x_{1}-R_{d}(C_{i}+x_{2})] \end{array}}\} \end{array}$$

where *x*_1_ = *J*/4 and *x*_2_ = 2Γ_*_; *C*_i_ is the intercellular CO_2_ level. According to our experimental data, *A*_j_-limitation in a light response curve of *Lilium* usually occurred at or below 1,000 μmol m^−2^ s^−1^, as a good linear relationship between *A*_n_ and *J* was observed within this range (Figure S4). The advantages of the NRH-A method over other existing methods including the most widely used variable-J method in deriving the average *g*_m_ was fully illustrated by Yin and Struik (2009a).

Equation (5) can also be applied to calculate *A*_c_ by setting: *x*_1_ = *V*_cmax_ and *x*_2_ = *K*_mC_(1+*O*/*K*_mO_). *V*_cmax_ was then estimated by fitting the combined Eqs. (1), (4) and (5) to the entire light response curve or *C*_i_ response curve using the already estimated values of *J*_max_, κ_2LL_, *R*_d_ and *g*_m_ as input.

### Temperature responses of photosynthesis parameters

To account for the effect of the varying temperature during measurement, temperature response functions were introduced so that the estimation of key parameters could be adjusted to the same reference temperature for the comparison among treatments. The temperature responses of *R*_d_ and Rubisco kinetic properties (*V*_cmax_, κ_mC_ and κ_mO_) were described by an Arrhenius function Equation (6), and the temperature responses of *J*_max_ and *g*_m_ were described by a peaked Arrhenius function Equation (7), normalized with respect to their values at 25 C:

(6) $$\begin{array}{r} X=X_{25}e^{\left(T-25\right)E_{x}/\left[298R\left(T+273\right)\right]} \end{array}$$

(7) $$\begin{array}{r} X=X_{25}e^{\left(T-25\right)E_{x}/\left[298R\left(T+273\right)\right]}\left[\frac{1+e^{\left(298S_{x}-D_{x}\right)/\left(298R\right)}}{1+e^{\left[\left(T+273\right)S_{x}-D_{x}\right]/\left[R\left(T+273\right)\right]}}\right] \end{array}$$

where *X* stands for each parameter; *X*_25_ is the value of each parameter at 25°C (*R*_d25_, *V*_cmax25_, κ_mC25_, κ_mO25_, *J*_max25_, and *g*_m25_); *E*_x_ is the activation energy of each parameter (*E*_Rd_, *E*_Vcmax_, *E*_KmC_, *E*_KmO_, *E*_Jmax_, and *E*_gm_); *S*_x_ and *D*_x_ are the entropy term and the deactivation energy, respectively (applying to *J*_max_ and *g*_m_); *T* is the leaf temperature; *R* is the universal gas constant. Since Rubisco kinetic properties are generally assumed conserved among C_3_ species (von Caemmerer et al., 2009), values of κ_mC25_, κ_mO25_, *E*_KmC_, and *E*_KmO_ were fixed at 272.4 μbar, 165.8 mbar, 80,990 J mol^−1^, and 23,720 J mol^−1^, respectively, according to Bernacchi et al. (2002). To avoid over-parameterization, *E*_Rd_ was fixed at 46,390 J mol^−1^ (Bernacchi et al., 2001); *S*_Jmax_ and *D*_Jmax_ were fixed at 650 J K^−1^ mol^−1^ (Harley et al., 1992) and 200,000 J mol^−1^ (Medlyn et al., 2002), respectively; *E*_gm_, *S*_gm_, and *D*_gm_ were fixed at 49,600 J mol^−1^, 1,400 J K^−1^ mol^−1^, and 437,400 J mol^−1^, respectively (Bernacchi et al., 2002).

### The relationships between biochemical parameters and leaf nitrogen content

The photosynthetic capacity parameters *V*_cmax25_ and *J*_max25_ are linearly related to *N*_a_ (Harley et al., 1992; Braune et al., 2009):

(8) $$\begin{array}{r} V_{cmax25}=\chi_{\text{V}}\left(N_{a}-N_{b}\right) \end{array}$$

(9) $$\begin{array}{r} J_{max25}=\chi_{\text{J}}\left(N_{a}-N_{b}\right) \end{array}$$

where *N*_b_ is the base leaf nitrogen content at or below which *A*_n_ is zero, and a value of 0.35 g N (m^2^ leaf)^−1^ was used in this study (Archontoulis et al., 2012); χ_V_ is the slope of *V*_cmax25_ against *N*_a_, and χ_J_ is the slope of *J*_max25_ against *N*_a_.

### Parameterization of the stomatal conductance model

A phenomenological model for stomatal conductance for CO_2_ transfer was first described by Ball et al. (1987), revised by Leuning (1995), and further revised by Yin and Struik (2009b). Li et al. (2012) called this model the BWB-Leuning-Yin model. In the model, stomatal conductance was described by:

(10) $$\begin{array}{r} g_{s}=g_{0}+\frac{A+R_{d}}{C_{i}-C_{i*}}f_{vpd} \end{array}$$

where *g*_0_ is the residual stomatal conductance when the irradiance approaches to zero; *C*_i*_ is the *C*_i_-based CO_2_ compensation point in the absence of *R*_d_ and was calculated as (Γ_*_−*R*_*d*_/*g*_*m*_) using Γ_*_, *R*_d_ and *g*_m_ calculated before as input; *f*_vpd_ is a function describing the effect of VPD, which is not yet understood sufficiently and may be described empirically as Yin and Struik (2009b):

(11) $$\begin{array}{r} f_{vpd}=\frac{1}{1/\left(a_{1}-b_{1}VPD\right)-1} \end{array}$$

where *a*_1_ represents the ratio of *C*_i_ to *C*_a_ for vapor saturated air, and *b*_1_ represents the decreasing slope of this ratio with increasing VPD, if *g*_0_ approaches to zero. Because of this obvious meaning of *a*_1_ and *b*_1_, we chose Equation (11), instead of the equation of Leuning (1995), for our analysis of the effect of VPD on *g*_s_. Combining Equations (10) and (11), *g*_0_, *a*_1_ and *b*_1_ can be estimated by using the data of *A*_n_, *C*_i_ and VPD obtained from gas exchange measurement. For that, measured stomatal conductance for water vapor transfer was divided by a factor 1.6 to convert it to *g*_s_ for CO_2_ transfer that is required for Equation (10).

### Statistical and model analyses

Using a non-linear regression with the GAUSS method in PROC NLIN of SAS (SAS Institute Inc., Cary, NC, USA), FvCB model parameters (*V*_cmax25_, *J*_max25_, κ_2LL_, *R*_d25_, *g*_m25_, *E*_Vcmax_, and *E*_Jmax_) and BWB-Leuning-Yin model parameters (*g*_0_, *a*_1_, and *b*_1_) were estimated. Whether or not the treatment effect on each estimated parameter was significant was tested using an *F*-test. Following that, conserved parameter values across treatment classes were also estimated.

With these estimated parameters available, we aimed to test to what extent conserved parameter values could be used to predict *A*_n_ and *g*_s_ under water and nitrogen stress combinations, for the purpose of simplifying model parameterization. For such, a step-wise procedure was followed. First, we analyzed whether or not water and nitrogen stress combinations change the linear relationships between biochemical parameters and *N*_a_, and tested to what extent conserved parameter values in the *C*_i_-based FvCB model (Equation 5) could be used to predict *A*_n_ under different water and nitrogen conditions. Second, we tested to what extent conserved parameter values could be used in the BWB-Leuning-Yin model to predict *g*_s_ under different water and nitrogen conditions. Third, we explored the coupled FvCB and BWB-Leuning-Yin model (for the analytical solution for this coupled model, see Yin and Struik, 2009b), which allows using *C*_a_ as input to predict *A*_n_. We used this coupled model to assess to what extent conserved parameter values in both the FvCB model and the BWB-Leuning-Yin model could be used to predict *A*_n_ (using *C*_a_ as input) across various water and nitrogen treatment regimes.

## Results

### Model parameterization

**Figure 1:**
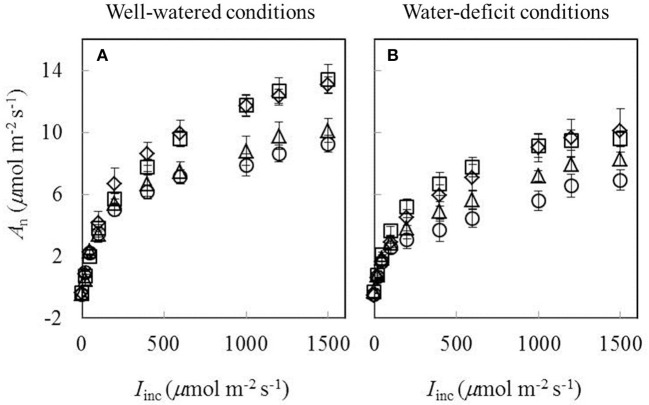
**Response curves of net CO_**2**_-assimilation rate (***A***_***n***_) to incident irradiance (***I***_**inc**_) obtained under (A)** well-watered conditions and **(B)** water-deficit conditions (N85: diamond; N65: square; N45: triangle; N25: circle. Mean ± standard error of 6 replicated plants). Leaf temperature during measurement = 20 ± 2°C.

**Figure 2:**
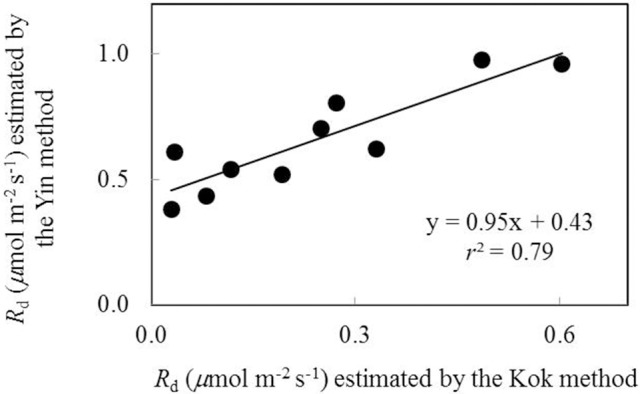
**The relationship between values of day respiration (***R***_***d***_) estimated by Kok and Yin methods (Each point represents the estimate of ***R***_***d***_ using the same ***A***_**n**_-***I***_**inc**_ curve)**.

Data of *A*_n_−*I*_inc_ curves showed that both water-deficit conditions and low nitrogen supply decreased *A*_n_ (Figure 1). The initial linear part of these curves was explored to estimate *R*_d_. Values of *R*_d_ estimated by the Kok method were generally lower than those estimated by the Yin method (Figure 2). The linear correlation between values of *R*_d_ estimated by the two methods (Figure 2) was used to correct all *R*_d_ estimated by the Kok method.

**Figure 3:**
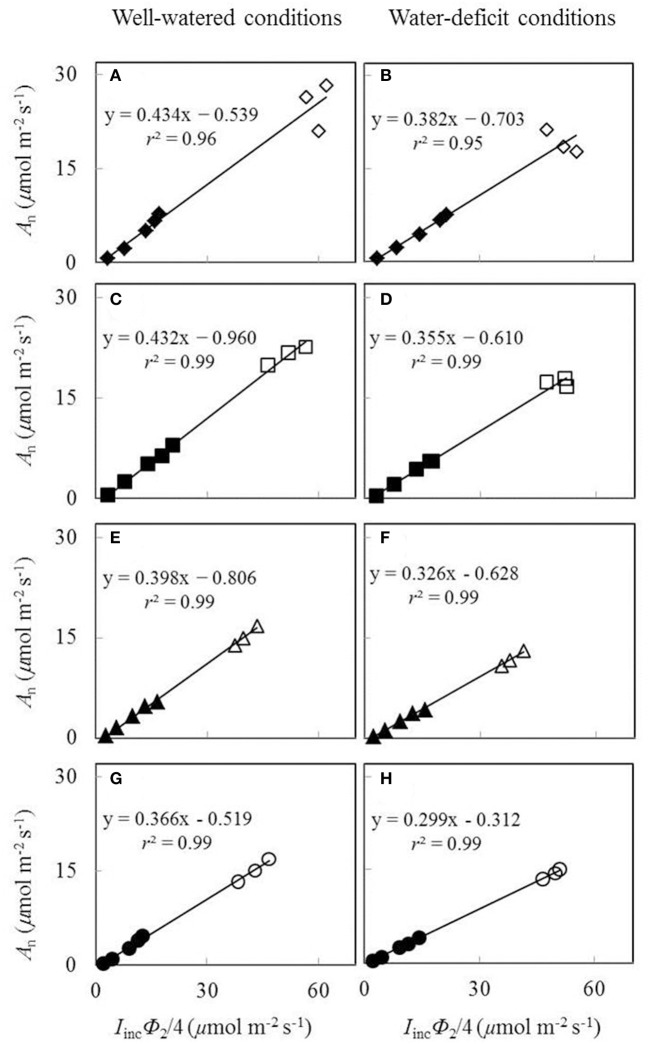
**Net CO_**2**_-assimialtion rate (***A***_**n**_), measured under a non-photorespiratory condition, as a function of ***I***_**inc**_Φ_**2**_/4 under well-watered conditions (A,C,E,G)** and water-deficit conditions **(B,D,F,H)** (N85: **A,B**; N65: **C,D**; N45: **E,F**; N25: **G,H**. Closed symbols are from low light levels of the *A*_n_-*I*_inc_ curves; open symbols are from three high CO_2_ levels at the same *I*_inc_ of 800 μmol m^−2^ s^−1^; data for open symbols and closed symbols in the same panel were measured on the same leaf; see the text).

The plot of *A*_j_ against (*I*_inc_ Φ_2_/4) using data obtained under low O_2_ condition from low light levels of the *A*_n_−*I*_inc_ curves and three high CO_2_ levels was essentially linear (Figure 3). Both water and nitrogen conditions affected the value of the linear slope *s*, the calibration factor used to convert Φ_2_ into *J*. The factor decreased by low nitrogen supply and by water-deficit conditions.

**Figure 4:**
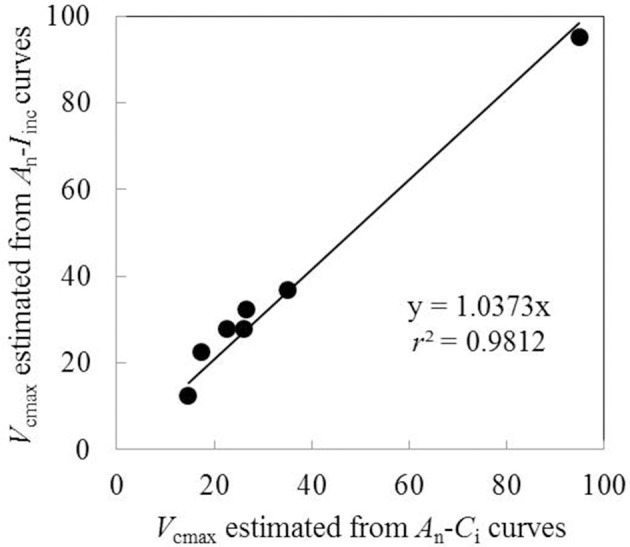
**Comparison of ***V***_**cmax**_ estimated from ***A***_**n**_-***I***_**inc**_ curves and ***A***_**n**_-***C***_**i**_ curves using the same input values of ***J***_**max**_, κ_**2*****LL***_, ***R***_***d***_ and ***g***_***m***_ (Each point represents value of ***V***_**cmax**_ estimated from ***A***_***n***_-***I***_**inc**_ curve or ***A***_***n***_-***C***_***i***_ curve measured on the same leaf)**.

*V*_cmax_ estimated from *A*_n_-*I*_inc_ curves and from available *A*_n_-*C*_i_ curves under the same measurement conditions were very similar when using the same input values of *J*_max_, κ_2LL_, *R*_d_, and *g*_m_ (Figure 4). This suggested the reliability of using *A*_n_-*I*_inc_ curves to estimate *V*_cmax_. The estimated parameter values of the FvCB model for each treatment are listed in Table 3, and those of the BWB-Leuning-Yin model and *g*_m_ are listed in Table 4. All parameters were reliably estimated, as the standard error values of the estimates were relatively small (Tables 3, 4).

**Table 3:** **List of parameter values (standard error of estimate in brackets if available) estimated for the FvCB model under different water and nitrogen treatments**.

**Treatment**	**κ_2LL_ (mol mol^−1^)**	***J*_max25_ (μmol m^−2^ s^−1^)**	***V*_cmax25_ (μmol m^−2^ s^−1^)**	***R*_d25_ (μmol m^−2^ s^−1^)**
**WELL-WATERED CONDITIONS**				
N85	0.242 (0.017)c	150 (6)a	109 (8)a	0.867 (0.18)a
N65	0.309 (0.020)a	141 (4)b	96 (5)b	0.696 (0.15)abc
N45	0.238 (0.013)cd	130 (5)c	90 (5)b	0.740 (0.12)ab
N25	0.251 (0.026)c	118 (6)d	77 (5)cd	0.492 (0.10)cd
**WATER-DEFICIT CONDITIONS**				
N85	0.218 (0.016)d	137 (8)bc	88 (6)bc	0.514 (0.17)bcd
N65	0.265 (0.017)bc	126 (3)cd	80 (5)c	0.448 (0.10)cd
N45	0.212 (0.029)d	103 (7)e	68 (4)d	0.412 (0.16)
N25	0.172 (0.019)e	96 (7)e	58 (4)e	0.409 (0.14)d

*Different letters following the data in the same column indicate significant difference (P < 0.05)*.

**Table 4:** **List of parameter values (standard error of estimate in brackets if available) estimated for parameters in the BWB-Leuning-Yin model of stomatal conductance (***g***_**s**_) and for mesophyll conductance (***g***_**m**_)**.

**Treatment**	***g***_**s**_			***g*_m_**
	***g*_0_ (mol m^−2^ s^−1^)**	***a*_1_ (-)**	***b*_1_ (kPa^−1^)**	***g*_m25_ (mol m^−2^ s^−1^ bar^−1^)**
**WELL-WATERED CONDITIONS**				
N85	0.021 (0.002)a	0.575 (0.029)b	0.203 (0.027)c	0.236 (0.017)a
N65	0.019 (0.002)a	0.671 (0.026)a	0.275 (0.021)b	0.197 (0.023)b
N45	0.014 (0.002)b	0.690 (0.033)a	0.321 (0.030)a	0.172 (0.032)bc
N25	0.011 (0.001)cd	0.688 (0.021)a	0.291 (0.021)ab	0.161 (0.035)bc
**WATER-DEFICIT CONDITIONS**				
N85	0.011 (0.001)cde	0.300 (0.041)c	0.013 (0.025)e	0.126 (0.014)cd
N65	0.009 (0.001)de	0.284 (0.039)c	0.007 (0.022)e	0.155 (0.025)c
N45	0.008 (0.001)e	0.308 (0.037)c	0.023 (0.024)e	0.103 (0.023)d
N25	0.012 (0.001)c	0.317 (0.034)c	0.086 (0.023)d	0.041 (0.013)e
**ESTIMATION OF OVERALL** ***a***_1_ **AND** ***b***_1_				
Well-watered conditions	–	0.661 (0.013)	0.270 (0.012)	–
Water-deficit conditions	–	0.262 (0.019)	0.013 (0.012)	–
All treatments	–	0.558 (0.012)	0.197 (0.010)	–

*Different letters following the data in the same column indicate significant difference (P < 0.05)*.

### The response of estimated parameter values to water and nitrogen treatments

Water-deficit conditions significantly decreased *V*_cmax25_, *J*_max25_, *k*_2LL_, and *R*_d25_ at all nitrogen levels (Table 3). *V*_cmax25_, *J*_max25_, and *R*_d25_ decreased with decreasing of nitrogen availability whereas κ_2LL_ showed such a response to a much less clear extent under both water-deficit conditions and well-watered conditions (Table 3). *V*_cmax25_, *J*_max25_, and κ_2LL_ were significantly lower in the combined water deficit and low nitrogen availability treatments than in other treatments (Table 3). Neither *E*_Jmax_ nor *E*_Vcmax_ was significantly affected by water and nitrogen treatments (Table S1).

Water-deficit conditions significantly decreased *g*_0_, *a*_1_, *b*_1_, and *g*_m25_ at all nitrogen levels (Table 4). *g*_0_ and *g*_m25_ decreased with decreasing nitrogen availability whereas *a*_1_ and *b*_1_ responded little to nitrogen treatments under both water-deficit conditions and well-watered conditions (Table 4). *g*_m25_ was significantly lower under the combined water deficit and the lowest nitrogen availability treatment than in other treatments (Table 4).

### The relationships between estimated parameter values and leaf nitrogen content

**Figure 5:**
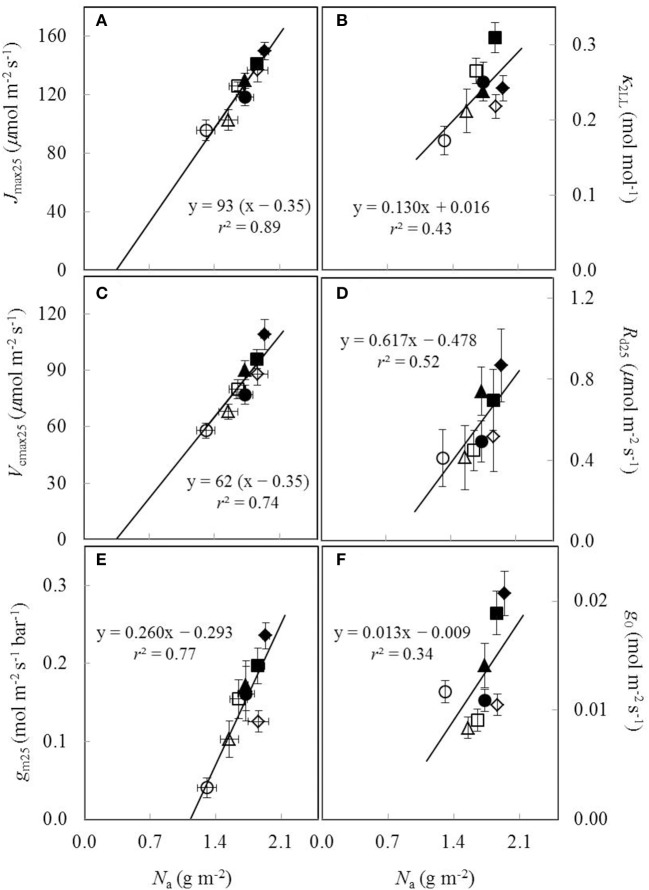
**The estimated parameters values for (A)** the maximum electron transport rate (*J*_max25_), **(B)** the conversion efficiency of limiting incident light into linear electron transport of photosystem II (κ_2LL_), **(C)** the maximum Rubisco carboxylation rate (*V*_cmax25_), **(D)** day respiration (*R*_d25_), **(E)** mesophyll conductance (*g*_m25_), and **(F)** residual stomatal conductance when the irradiance approaches to zero (*g*_0_), all as a function of leaf nitrogen content (*N*_a_) under different water and nitrogen treatments (Well-watered conditions: closed symbols; water-deficit conditions: open symbols. N85: diamond; N65: square; N45: triangle; N25: circle. Vertical error bar indicates standard error of estimate; horizontal error bar indicates standard error of the mean measured value).

**Figure 6:**
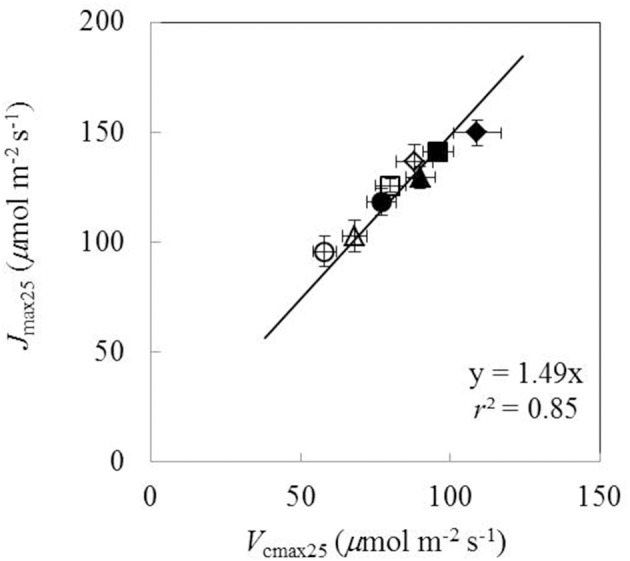
**The relationship between the maximum Rubisco carboxylation rate (***V***_**cmax25**_) and the maximum electron transport rate (***J***_**max25**_) under different water and nitrogen treatments (Well-watered conditions: closed symbols; water-deficit conditions: open symbols**. N85: diamond; N65: square; N45: triangle; N25: circle. Error bars indicate standard error of estimate).

Under both water-deficit conditions and well-watered conditions, *V*_cmax25_, *J*_max25_, κ_2LL_, *R*_d25_, *g*_m25_, and *g*_0_ linearly increased with increasing *N*_a_ (Figure 5). *X*_V_ and *X*_J_ were determined as 62 μmol (g N)^−1^ s^−1^ and 93 μmol (g N)^−1^ s^−1^, respectively (Figures 5A,C). The *N*_a_-dependent relationship was relatively less clear for other parameters (Figures 5B,D-F), but an *F*-test revealed that water and nitrogen treatments did not significantly alter the linear relationships in all the six parameters. Linear relationship existed between *V*_cmax25_ and *J*_max25_ with a slope of 1.49 under different water and nitrogen treatments (Figure 6).

### Comparison between model predictions and measured values for *A_n_* and *g_s_*

**Figure 7:**
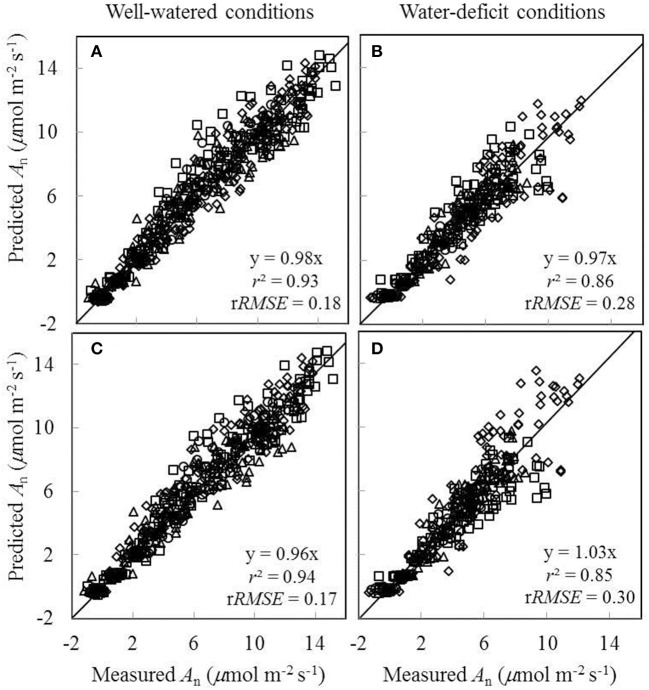
**Comparisons between the measured net CO_**2**_-assimilation rate (***A***_***n***_) and the predicted ***A***_***n***_ by the ***C***_***i***_-based FvCB model either using treatment-specific parameter values (A,B)**, or using shared parameter values **(C,D)** (Well-watered conditions: **A,C**; water-deficit conditions: **B,D**. N85: diamond; N65: square; N45: triangle; N25: circle). The equation in each panel represents the linear regression of predicted (y) vs. measured values (x) by forcing the line through the origin, *r*^2^ is the determination coefficient of the regression, and r*RMSE* is the relative root-mean-square error (= $\;\frac{1}{\bar{x}}\sqrt{\frac{\sum_{i\;=\;1}^{n}{\left(y_{i}-x_{i}\right)}^{2}}{n}}$, where *n* is the number of data points, and $\bar{x}$ is the mean of the measured values).

Since the linear relationships between biochemical parameters and *N*_a_ were found to exist under different treatment combinations (Figure 5), we further tested to what extent conserved parameter values could be used in the FvCB model to predict *A*_n_ under different water and nitrogen conditions. Two sets of comparisons between the measured *A*_n_ and the predicted *A*_n_ were conducted, (i) using treatment-specific parameter values (i.e., using specific parameter values obtained under each treatment) (Figures 7A,B), and (ii) using shared parameter values (i.e., incorporating the *N*_a_-dependent linear relationships and using overall *E*_Jmax_ and *E*_Vcmax_ values) (Figures 7C,D). For this second set of comparison, the overall values of *E*_Jmax_ and *E*_Vcmax_ for all treatments were estimated (Table S1) by incorporating the linear relationships between parameters (*V*_cmax25_, *J*_max25_, κ_2LL_, *R*_d25_, and *g*_m25_) and *N*_a_. The coefficient of determination (*r*^2^) between estimated and measured *A*_n_ in both comparisons ranged from 0.85 to 0.94 (Figure 7).

**Figure 8:**
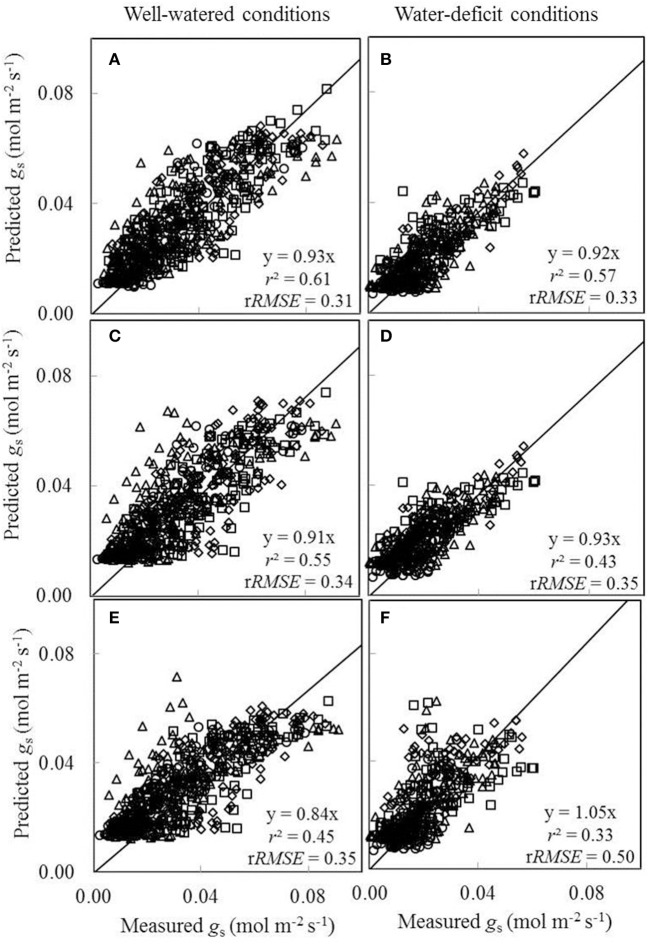
**Comparisons between the measured stomatal conductance for CO_**2**_ diffusion (***g***_***s***_) and the predicted ***g***_***s***_ by the BWB-Leuning-Yin model either using treatment-specific parameter values (A,B)**, or using shared parameter values for each water treatment **(C,D)**, or using shared parameter values for all treatments **(E,F)** (Well-watered conditions: **A,C,E**; water-deficit conditions: **B,D,F**. N85: diamond; N65: square; N45: triangle; N25: circle). For further details, see Figure 6.

We also tested to what extent conserved parameter values could be used in the BWB-Leuning-Yin model (Equations 10 and 11) to predict *g*_s_ under different water and nitrogen conditions. Since nitrogen had been found to have little effect on *a*_1_ and *b*_1_ (Table 4) and *g*_0_ could be linearly correlated with *N*_a_ under both well-watered conditions and water-deficit conditions (Figure 5F), we tested to what extent conserved values of *a*_1_, *b*_1_, and *g*_0_ can be used. For this purpose, we incorporated the linear relationships between model parameters (*g*_0_ and *g*_m25_) and *N*_a_, and estimated overall values of *a*_1_ and *b*_1_ for all treatments (Table 4). Three sets of comparisons between the measured *g*_s_ and the predicted *g*_s_ were conducted, (i) using treatment-specific parameter values (Figures 8A,B), (ii) using shared parameter values for each water treatment (i.e., incorporating the *N*_a_-dependent linear relationships and using overall values of *a*_1_ and *b*_1_ for each water treatment group given in Table 4) (Figures 8C,D), and (iii) using shared parameter values for all treatments (i.e., incorporating the *N*_a_-dependent linear relationships and using overall values of *a*_1_ and *b*_1_ for all treatments given Table 4) (Figures 8E,F). Using treatment-specific parameter values in the BWB-Leuning-Yin model, the *r*^2^ between estimated and measured *g*_s_ was 0.61 under well-watered conditions and 0.57 under a water deficit (Figures 8A,B); using shared parameter values for each water treatment, the *r*^2^ was 0.55 for well-watered plants and 0.43 under a water deficit (Figures 8C,D). When shared parameters were used for all treatments, *g*_s_ was appreciably underestimated under well-watered conditions (Figure 8E), but overestimated under a water deficit (Figure 8F). This third set of predictions of *g*_s_, when compared with the first set of predictions, underestimated *g*_s_ by 9% under well-watered conditions and overestimated *g*_s_ by 13% under water-deficit conditions.

**Figure 9:**
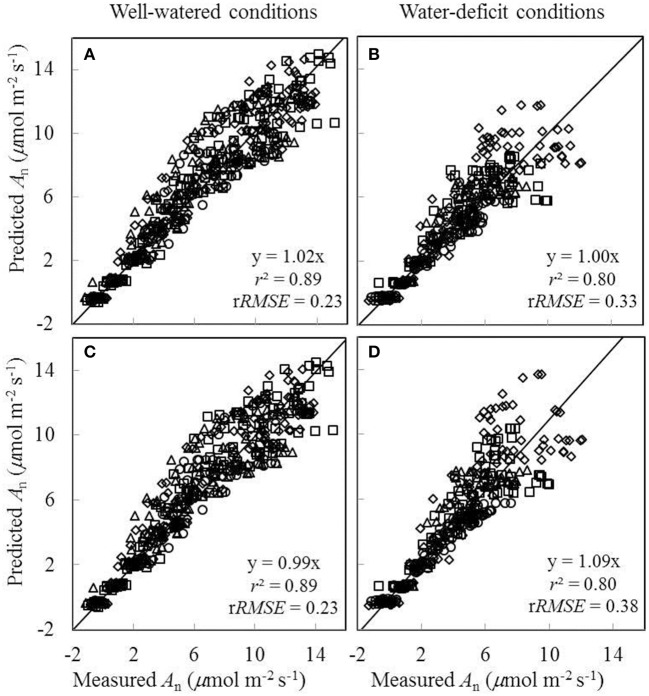
**Comparisons between the measured net CO_**2**_-assimilation rate (***A***_***n***_) and the predicted ***A***_***n***_ by the coupled FvCB and BWB-Leuning-Yin model using shared values of the FvCB model parameters for all treatments combined either with shared values of the BWB-Leuning-Yin model parameters for each water treatment (A,B)**, or with shared values of the BWB-Leuning-Yin model parameters for all treatments **(C,D)** (Well-watered conditions: **A,C**; water-deficit conditions: **B,D**. N85: diamond; N65: square; N45: triangle; N25: circle). For further details, see Figure 6.

As *g*_s_ was either underestimated or overestimated by the BWB-Leuning-Yin model using shared parameter values for all treatments (Figures 8E,F), we further assessed the impact of this inaccurate estimation of *g*_s_ on the prediction of *A*_n_. Two sets of comparisons between the measured *A*_n_ and the predicted *A*_n_ were conducted. In the first comparison, shared values of the FvCB model parameters for all treatments and shared values of the BWB-Leuning-Yin model parameters for each water treatment were used in the coupled model; the *r*^2^ between estimated and measured *A*_n_ was 0.89 under well-watered conditions and 0.80 under water-deficit conditions (Figures 9A,B). In the second comparison, shared values of both the FvCB model parameters and the BWB-Leuning-Yin model parameters for all treatments were used; the *r*^2^ was 0.89 under well-watered conditions (Figure 9C), but *A*_n_ was overestimated by 9% under water-deficit conditions (Figure 9D).

## Discussion

### Methodology to estimate photosynthetic parameters

In our study, all model parameters were estimated based on the *A*_n_-*I*_inc_ curves, instead of *A*_n_-*C*_i_ curves, for estimating the FvCB parameters. We tested that the estimated *V*_cmax_ values by using these two types of curves were quite similar (Figure 4), as also shown in a previous study (Archontoulis et al., 2012). The approach of using *A*_n_-*I*_inc_ curves provides an alternative to the prevailing approach of using *A*_n_-*C*_i_ curves and has its own advantages. First, the FvCB model is commonly used to predict leaf photosynthesis in canopies under field conditions, where it is the light level, not the CO_2_ level, that fluctuates most significantly in space and in time. This suggests that the FvCB parameters estimated from *A*_n_-*I*_inc_ curves should more closely represent field situations, relative to those based on *A*_n_-*C*_i_ curves. Second, using *A*_n_-*C*_i_ curve is known to have problems of CO_2_ leakage and down-regulation of Rubisco at the low level of CO_2_ during the measurement. The *A*_n_-*I*_inc_ curve-based approach avoids these problems since the whole response curve is measured under ambient CO_2_ level. However, using *A*_n_-*I*_inc_ curves also tends to have problems. First, *V*_cmax_ cannot always be estimated from *A*_n_-*I*_inc_ curves since the entire *A*_n_-*I*_inc_ curve can be *A*_j_ limited sometimes (Archontoulis et al., 2012), especially for field crops that have high light saturating point (e.g., cotton, Wise et al., 2004). Second, the rate of TPU (triose phosphate utilization), if exerting a limitation on photosynthesis, cannot be estimated using *A*_n_-*I*_inc_ curves since like Rubisco limitation, any TPU limitation on *A*_n_-*I*_inc_ curves also happens at high irradiance levels (Archontoulis et al., 2012). Nevertheless, our limited data (Figure 4) show the evidence in support of using *A*_n_-*I*_inc_ curves as an alternative approach to estimate *V*_cmax_. More comparisons between the two approaches using *A*_n_-*I*_inc_ and using *A*_n_-*C*_i_ curves are needed for different crop types and environments.

We adopted some parameter values from the literature as input to avoid over-parameterization of the FvCB model. First, θ (the convexity factor for response of electron transport rate to incident light) was set to a constant value of 0.8 according to Yin and Struik (2015). It is worthy to notice that the actual value of θ could vary across species and environments. In our experiment, θ may be affected by different water and nitrogen treatments, as well as different light environment caused by different growth season. Initial analyses showed that letting θ be fitted as well resulted in enormous unrealistic variation of the estimated *J*_max_ and κ_2LL_. Since the biological meaning of θ is less obvious than that of *J*_max_ and κ_2LL_, we decided to set θ as a constant value to avoid biased estimations of *J*_max_ and κ_2LL_. Equation (4) with θ of 0.8 generates a very similar light response shape as given by the other widely used quadratic hyperbolic equation initially used by Harley et al. (1992). Second, in line with some previous studies (Xu and Baldocchi, 2003; Li et al., 2012), we adopted the activation energy of *R*_d_ (*E*_Rd_) and *g*_m_ (*E*_gm_), the deactivation energy of *J*_max_ (*D*_Jmax_) and *g*_m_ (*D*_gm_), and the entropy term of *J*_max_ (*S*_Jmax_) and *g*_m_ (*S*_gm_) from literature (Bernacchi et al., 2001, 2002). Whether or not these temperature response parameters change with water and nitrogen conditions is still not clear and further studies are needed. Third, Rubisco kinetic properties (κ_mC25_, κ_mO25_, *E*_KmC_, and *E*_KmO_) were adopted from Bernacchi et al. (2002). Despite the generally assumption that Rubisco kinetic properties are conserved among C_3_ species (von Caemmerer et al., 2009), values of these constants reported in the literature are different (Bernacchi et al., 2001, 2002; Dreyer et al., 2001). The choice of Rubisco parameters also affected our FvCB parameter estimation. Since all parameters in the FvCB model are interrelated with each other, potential errors in our parameter estimation exist if parameter values we adopted from the literature were not applicable in our study.

### Photosynthetic biochemical parameters in response to water and nitrogen conditions

Our study showed that a long-term mild water deficit and water and nitrogen stress combinations did not have significant effects on the linear relationships between biochemical parameters of the FvCB model (i.e., *J*_max25_, κ_2LL_, *V*_cmax25_) and leaf nitrogen content per unit area (*N*_a_) (Figure 5). Previous studies showed that a short-term water deficit did not change the linear relationships between biochemical parameters and *N*_a_ (Díaz-Espejo et al., 2006; Gu et al., 2012), whereas under long-term drought, either the slopes of the relationships between biochemical parameters and *N*_a_ were changed (Wilson et al., 2000; Díaz-Espejo et al., 2006) or considering the effect of leaf mass per area (LMA) in the linear regressions was needed (Xu and Baldocchi, 2003). A few other studies (Damour et al., 2008, 2009) found that drought totally modified the fundamental relationships between *J*_max_ and *N*_a_ since *N*_a_ was either increasing (Damour et al., 2008) or not affected (Damour et al., 2009) under drought whereas *J*_max_ decreased. The discrepancy of the response of *N*_a_ to drought found in different studies may be caused by different species. Damour et al. (2008) worked with lychee tree and Damour et al. (2009) worked with mango tree, whereas we focused on herbaceous species *Lilium* and Gu et al. (2012) worked with rice. Besides, different approaches used to estimate FvCB parameters could also affect the results in different studies. First, as stated earlier, we used *A*_n_-*I*_inc_ curves to parameterize the FvCB model. Whether or not the approach of using *A*_n_-*I*_inc_ curves and the approach of using *A*_n_-*C*_i_ curves yield similar results under drought still requires more comparisons. Second, early studies tend to ignore *s* (the calibration factor for converting fluorescence-based efficiency of photosystem II photochemistry Φ_2_ into electron transport rate *J*) and *g*_m_ (mesophyll conductance) during the estimation of biochemical parameters. This could lead to inaccurate estimation of biochemical parameters since both *s* (Figure 3) and *g*_m_ (Table 4; also reviewed in Flexas et al., 2008) decreased under drought.

The calibration factor *s* used to convert Φ_2_ into *J* is actually a lumped physiological parameter (*s* = ρ_2_β[1–*f*_pseudo_/(1−*f*_cyc_)]) that includes the absorptance of light by leaf photosynthetic pigments (β), the proportion of absorbed light partitioned to photosystem (PS) II (ρ_2_), and the fraction of electrons at PSI following the cyclic transport around PSI (*f*_cyc_) and following the pseudocyclic transport (*f*_pseudo_) (Yin et al., 2009; Yin and Struik, 2009a). *s* was found to decrease by low nitrogen supply in previous study (Yin et al., 2009), which is also found in our study (Figure 3). This decrease may be explained by the decreasing of β as a result of the decreased photosynthetic pigments in low-nitrogen leaves (Evans and Terashima, 1987). Interestingly, we found that *s* was smaller under water-deficit conditions compared to that under well-watered conditions despite the similar *N*_a_ (e.g., *s* in N65 under well-watered conditions compared with *s* in N85 under water-deficit conditions). It has been reported that drought did not change the partitioning of electrons between PSI and PSII (Genty et al., 1987). However, stomatal closure caused by drought results in the decreasing of CO_2_ concentration in the leaf, and consequently the amount of electrons used for CO_2_ fixation decreases (Cornic and Briantais, 1991). Excessive electrons need to be consumed by other sinks apart from CO_2_ fixation by following pseudo-cyclic electron transport (Cornic and Briantais, 1991; Biehler and Fock, 1996), or electrons need to follow cyclic flow around PSI (Kohzuma et al., 2009). Our results for the decreased *s* under water-deficit conditions independent on *N*_a_ suggest that drought induced an increase of *f*_pseudo_ or *f*_cyc_ or both in our experimental conditions.

Associated with estimating the factor *s*, mitochondrial day respiration (*R*_d_) was estimated. Water-deficit conditions did not affect *R*_d_ in all N treatments, and there were non-significant effects of nitrogen on *R*_d_ under both well-watered conditions and water-deficit conditions (Table 3). Nevertheless, water-deficit conditions significantly decreased *R*_d_ in N85 and N45 treatments and generally there was a trend showing that drought and decreasing of nitrogen level decreased *R*_d_ (Table 3), as also revealed in some previous studies (González-Meler et al., 1997; Huang and Fu, 2000). Therefore, we established an *N*_a_-dependent relationship of *R*_d_ (Figure 5F) and applied this relationship to capture the changes of *R*_d_ under different water and nitrogen conditions. The linear relationship between respiration rate and leaf nitrogen content was also found under different light conditions (Ryan, 1995) and growth locations (Reich et al., 1998).

A relatively stable *J*_max25_/*V*_cmax25_ ratio among different water and nitrogen treatments was found in our study (Figure 6), in line with some previous studies (Makino et al., 1992; Walcroft et al., 1997; Díaz-Espejo et al., 2006). Some studies simplified the parameterization of the FvCB model by using a fixed value for either the *J*_max_/*V*_cmax_ ratio (Kosugi et al., 2003) or the *J*_max25_/*V*_cmax25_ ratio (Müller et al., 2005). However, care needs to be taken in setting a constant *J*_max_/*V*_cmax_ ratio. First, when temperature varies, this ratio cannot be constant because *J*_max_ and *V*_cmax_ have different temperature response curves. In fact, the *J*_max_/*V*_cmax_ ratio was found to decrease with temperature increase (Walcroft et al., 1997; Medlyn et al., 2002; Díaz-Espejo et al., 2006). When scaled to a common temperature, a better correlation between *J*_max_ and *V*_cmax_ was found (Leuning, 1997). Second, *g*_m_ has a strong influence on this *J*_max_/*V*_cmax_ ratio. In early studies (Grassi et al., 2002) when *g*_m_ was not considered, a *J*_max_/*V*_cmax_ ratio of ca 2.0 was obtained (Leuning, 1997), which is higher than our estimate where *g*_m_ was considered (ca 1.5, Figure 6). Finally, some studies found that water and nitrogen conditions also affected the *J*_max_/*V*_cmax_ ratio (Grassi et al., 2002; Gu et al., 2012). Therefore, the approach using a fixed value for the *J*_max_/*V*_cmax_ ratio to parameterize the FvCB model should receive critical reservation (Xu and Baldocchi, 2003; Archontoulis et al., 2012).

In short, our study suggested that it is feasible to incorporate linear relationships between biochemical parameters and *N*_a_ in the FvCB model to predict photosynthesis under different water and nitrogen conditions, since the FvCB model using shared parameter values for all treatments gave satisfactory predictions of *A*_n_ under different water and nitrogen conditions (Figures 7C,D).

### Stomatal conductance parameters and mesophyll conductance in response to water and nitrogen conditions

Accurately modeling stomatal conductance (*g*_s_) and mesophyll conductance (*g*_m_) are necessary steps toward predicting *A*_n_ under changing environments. The BWB-type model of *g*_s_ takes into account the effects of both environments and plant physiological status on *g*_s_, and has been widely tested able to satisfactorily predict *g*_s_ for well-watered plants (Leuning, 1995; Li et al., 2012). Some efforts have been devoted to predict *g*_s_ under drought conditions using the BWB-type model by introducing proper approaches to adjust parameter values used in the model. In general, most studies kept *g*_0_ (residual stomatal conductance when the irradiance approaches to zero) as a fixed value and adjusted the value for the slope (roughly represents *a*_1_ and *b*_1_ in the BWB-Leuning-Yin model used in our study) by introducing a modifying factor of soil moisture (Egea et al., 2011; Li et al., 2012), or precipitation and evaporation (Baldocchi, 1997), or predawn xylem water potential (Sala and Tenhunen, 1996), or leaf nitrogen content and leaf water potential (Müller et al., 2014). Leuning (1995) suggested that the BWB-type model should be able to predict *g*_s_ under water-deficit conditions by only adjusting the value for *a*_1_. We found that both *a*_1_ and *b*_1_ decreased with the decreasing of SWP (Table 4), and without considering these decreases, *g*_s_ was overestimated under water-deficit conditions (Figure 8F). Further estimation of *a*_1_ under water-deficit conditions by using the value for *b*_1_ obtained under well-watered conditions resulted in a value of 0.586 for *a*_1_, which is much larger than the original value of 0.262 obtained under water-deficit conditions (Table 4). Therefore, values for both *a*_1_ and *b*_1_ need to be adjusted to properly predict *g*_s_ under water-deficit conditions. However, *a*_1_ and *b*_1_ were little affected by nitrogen availability (Table 4) and no correlation between *a*_1_ and *N*_a_, nor between *b*_1_ and *N*_a_, under different water and nitrogen conditions was found in our study. The approach introducing a modifying factor of leaf nitrogen content on the slope (Müller et al., 2014) is able to predict *g*_s_ in response to drought, and this could merely be due to similar responses of leaf nitrogen content and the slope to soil water condition rather than because a functional relationship exists between the slope and leaf nitrogen content. Our study did not present a quantitative relationship of *a*_1_ and *b*_1_ with water supply conditions since there were only two water-level treatments. Further studies including more water levels would be needed to quantify changes of *a*_1_ and *b*_1_ under different water and nitrogen conditions.

*g*_0_ was affected by both water conditions and nitrogen availability (Table 4), and a linear relationship between *g*_0_ and *N*_a_ (Figure 5F) was used in our study to take into account the changes of *g*_0_ under different water and nitrogen conditions. Although this linear relationship is less clear compared to linear relationships between *N*_a_ and biochemical parameters (e.g., *J*_max25_ and *V*_cmax25_) (Figure 5), an *F* test showed that there is no significant difference between using a conserved linear relationship and using separate relationships to describe the *N*_a_ dependence of *g*_0_ in response to water-deficit conditions. Under drought, plants tend to reserve water by reducing water loss, which makes it unlikely that *g*_0_ is unaffected by water-deficit conditions. However, few modeling studies considered the change of *g*_0_ under drought condition (Misson et al., 2004; Keenan et al., 2010). The reason for using a fixed value for *g*_0_ in previous studies could be that changing the value of *g*_0_ should not affect the prediction of *g*_s_ very much for plants with relatively high *g*_s_ since the value of *g*_0_ itself is normally very small and approaches to zero. However, this may not hold true for plants with low *g*_s_, as is the case in our study, since the value of *g*_0_ may have relatively larger impact on predicting *g*_s_.

*g*_m_ has received growing attentions in modeling photosynthesis (Niinemets et al., 2009), since *g*_m_ has been found to be finite and vary greatly among environments (Flexas et al., 2008; Yin et al., 2009). Previous studies found that *g*_m_ decreased under drought and low nitrogen availability (reviewed in Flexas et al., 2008). We found that *g*_m_ was enhanced by high nitrogen level and strongly decreased by the combination of water deficit and low nitrogen availability (Table 4). A relatively strong linear correlation between *g*_m_ and *N*_a_ was found in our study (Figure 5E), as also found in previous studies (von Caemmerer and Evans, 1991; Warren, 2004). Such a correlation may be explained by the surface area of the chloroplasts facing the cell walls, an anatomical determinant of *g*_m_ (von Caemmerer and Evans, 1991; Evans et al., 1994), which depends on *N*_a_.

Our results showed that the relation between *g*_m_ and *N*_a_ was hardly changed by water-deficit conditions (Figure 5E). In contrast, Gu et al. (2012) found that the change of *g*_m_ by water-deficit conditions was not explained by the change of *N*_a_ but was negatively correlated with LMA. Nevertheless, LMA is generally considered as setting a limitation for the maximum *g*_m_ (Flexas et al., 2008; Perez-Martin et al., 2009) rather than is used to model *g*_m_ in response to environments, mainly because the change of LMA results from the long-term environmental adaptation of the plants (Poorter et al., 2009) whereas *g*_m_ can vary quickly in response to environmental changes (Flexas et al., 2006). This is supported by our result of using the *N*_a_-dependent linear relationship to take into account the effects of water and nitrogen on *g*_m_. Together with the incorporation of other *N*_a_-dependent relationships of biochemical parameters, the model yielded similar results of *A*_n_ prediction compared to those using treatment-specific parameter values (Figure 7).

Some studies incorporated a dependence of *g*_m_ on *g*_s_ in the photosynthesis model (Cai et al., 2008) as a close correlation between *g*_s_ and *g*_m_ in response to soil water deficit was commonly observed (Flexas et al., 2002; Warren, 2008; Perez-Martin et al., 2009). An approach incorporating the dependence of *g*_m_ on *g*_s_ was shown to give better prediction of *A*_n_ of different genotypes than the one incorporating the dependence of *g*_m_ on leaf nitrogen (Ohsumi et al., 2007). However, the approach has been criticized as having no physiological justification (Niinemets et al., 2009) since *g*_m_ and *g*_s_ respond differently to other environmental factors such as VPD (Warren, 2008; Perez-Martin et al., 2009). As there is not yet sufficient physiological knowledge to reliably quantify the variability of *g*_m_, some studies merely used a modifying factor of soil water conditions to take into account the effect of water deficit on *g*_m_ (Keenan et al., 2010; Egea et al., 2011). Whether or not the linear relationship between *g*_m_ and *N*_a_ could be a promising step toward modeling the variation of *g*_m_ needs to be further tested.

### The effect of *g_s_* estimation on the prediction of *A*_n_

The coupled FvCB and BWB model has been increasingly used to model photosynthesis in response to environmental changes such as elevated CO_2_ (Harley et al., 1992) and drought stress (Keenan et al., 2010; Müller et al., 2014) and seasonal changes (Kosugi et al., 2003). Normally in those previous studies, values of the biochemical parameters were related to the leaf nitrogen content and values of the stomatal conductance model parameters were changed according to the CO_2_ level (Harley et al., 1992), leaf water potential (Müller et al., 2014), or growth season (Kosugi et al., 2003).

Our study showed that considering the decreases of the stomatal conductance model parameters (*a*_1_ and *b*_1_) by drought was needed, otherwise, the coupled FvCB and BWB-Leuning-Yin model overestimated *A*_n_ under drought (Figure 9D) due to an overestimation of *g*_s_ (Figure 8F). The strong decrease of *a*_1_ by drought (Table 4) indicates the decreasing of *C*_i_/*C*_a_ ratio for vapor saturated air. The decrease of *b*_1_ by drought (Table 4) suggests a negligible control of VPD on *g*_s_ under drought condition. These results are in line with previous studies that under drought condition, *g*_s_ at vapor nearly saturated air tended to be lower and *g*_s_ was less sensitive to VPD (Forseth and Ehleringer, 1983; Perez-Martin et al., 2009). However, an exceptional case, which *g*_s_ showed much stronger sensitivity to VPD under drought, was also found in the previous study without an explanation provided (Perez-Martin et al., 2009).

The BWB-Leuning-Yin model without considering the effect of water level on *a*_1_ and *b*_1_ also underestimated *g*_s_ under well-watered conditions (Figure 8E). But the subsequent prediction of *A*_n_ was not affected much (Figure 9C). This is probably explained by that under well-watered conditions, *C*_i_ is generally high and changing *C*_i_ at its high level only slightly affects *A*_n_ according to the diminishing-return relationship of *A*_n_ vs. *C*_i_. Therefore, as shown in Figure 9, the estimation of *g*_s_ had more effect on the prediction of *A*_n_ under water-deficit conditions than under well-watered conditions.

## Concluding remarks

A previous analysis (Yin, 2013) showed that the relationship of many crop model parameters (including those FvCB biochemical parameters) as a function of plant nitrogen status was little altered by elevated CO_2_ concentration. Our present study examined whether this assertion could be extended for the water and nitrogen stress combinations. We showed that the *N*_a_ dependence of biochemical parameters of the FvCB model, *g*_0_ of the BWB-Leuning-Yin model and the *g*_m_ value were little altered by water and nitrogen stress combinations (Figure 5). By incorporating these *N*_a_-dependent relationships with the FvCB model and BWB-Leuning-Yin model, parameterization of these models could be simplified while maintaining satisfactory predictions. The obvious exception is parameters *a*_1_ and *b*_1_ of the BWB-Leuning-Yin model, which depended little on nitrogen treatments but greatly on water treatments (Table 4). This is probably because the BWB-Leuning-Yin model is largely phenomenological, and its related conclusions are only valid for the specific species and conditions examined in this study. While the variation of parameters *a*_1_ and *b*_1_ had a great impact on the prediction of stomatal conductance, it had a considerably lower impact on the prediction of leaf photosynthesis. Nevertheless, a further study is needed to quantify how these two parameters vary with water-deficit conditions, as they have a stronger bearing on modeling leaf transpiration.

## Author contributions

NZ analyzed the data and drafted the manuscript. GL, SY, DA, and QS carried out the measurements. WL and XY made substantial contributions to conception and experimental design, and critically revised the manuscript.

### Conflict of interest statement

The authors declare that the research was conducted in the absence of any commercial or financial relationships that could be construed as a potential conflict of interest. The reviewer DT and handling Editor declared their shared affiliation, and the handling Editor states that the process nevertheless met the standards of a fair and objective review.

## Supplementary material

Table S1 **Activation energy of ***J***_**max**_ and ***V***_**cmax**_ (standard error of estimate in brackets) estimated for each water and nitrogen treatments and their shared values for all treatments**. Different letters following the data in the same column indicate significant difference (*P* < 0.05).

Figure S1 **Daily mean, maximal and minimal air temperature at the height of 1.5 m above ground inside the greenhouse during (A)** Exp. 1, **(B)** Exp. 2, **(C)** Exp. 3, and **(D)** Exp. 4. Solid curve is daily mean air temperature, dashed curve on top is the daily maximal air temperature, dotted curve at bottom is the daily minimal air temperature.

Figure S2 **Vapor pressure deficit (VPD) at the height of 1.5 m above ground inside the greenhouse during (A)** Exp. 1, **(B)** Exp. 2, **(C)** Exp. 3, and **(D)** Exp. 4. Curve is daily mean VPD, solid line is the average daily mean VPD during the whole growth period, and dashed lines are the maximal and minimal daily mean VPD during the whole growth period, respectively.

Figure S3 **Daily mean photosynthetic active radiation (PAR) above crop canopy inside the greenhouse during (A)** Exp. 1, **(B)** Exp. 2, **(C)** Exp. 3, and **(D)** Exp. 4. Curve is daily mean PAR, and line is the average daily mean PAR during the whole growth period.

Figure S4 **Relationships between ***A***_**n**_ and ***J*** under well-watered conditions (A,C,E,G)** and water-deficit conditions **(B,D,F,H)** (N85: **A,B**; N65: **C,D**; N45: **E,F**; N25: **G,H**). All data points were chosen from light levels at or below 1,000 μmol m^−2^ s^−1^ and leaf temperature at 20 ± 2°C (Vertical error bar indicates standard error of measured *A*_n_; horizontal error bar indicates standard error of calculated *J*).
