# Supplementary material for: Can the Responses of Photosynthesis and Stomatal Conductance to Water and Nitrogen Stress Combinations Be Modeled Using a Single Set of Parameters?
Source: Front Plant Sci. 2017 Mar 28;8:328. doi: 10.3389/fpls.2017.00328 (PMC5368885; doi:10.3389/fpls.2017.00328)
Supplement: Table S1 — Activation energy of Jmax and Vcmax (standard error of estimate in brackets) estimated for each water and nitrogen treatments and their shared values for all treatments. Different letters following the data in the same column indicate significant difference (P < 0.05). [file Table1.docx]

**Supplementary table**

Table S1. Activation energy of *J*_max_ and *V*_cmax_ (standard error of estimate in brackets) estimated for each water and nitrogen treatments and their shared values for all treatments. Different letters following the data in the same column indicate significant difference (P<0.05).

| Treatment | *E*_Jmax_ (J mol^-1^) | *E*_Vcmax_ (J mol^-1^) |
| --- | --- | --- |
| Well-watered conditions | | |
| N85 | 54618 (6862) ab | 57874 (8377) a |
| N65 | 57737 (3539) a | 47231 (6745) ab |
| N45 | 48441 (4122) b | 41964 (5920) b |
| N25 | 46384 (5581) b | 41749 (6870) b |
| Water-deficit conditions | | |
| N85 | 51658 (6275) ab | 48296 (9459) ab |
| N65 | 49017 (2519) b | 43407 (8421) ab |
| N45 | 49854 (6782) b | 42842 (7082) ab |
| N25 | 47369 (6782) b | 46310 (8884) ab |
| Estimation of overall *E*_Jmax_ and *E*_Vcmax_ shared for all treatments | | |
| -- | 52083 (1040) | 45909 (2291) |
