# Supplementary figures and images for: Can the Responses of Photosynthesis and Stomatal Conductance to Water and Nitrogen Stress Combinations Be Modeled Using a Single Set of Parameters?
Source: Front Plant Sci. 2017 Mar 28;8:328. doi: 10.3389/fpls.2017.00328 (PMC5368885; doi:10.3389/fpls.2017.00328)

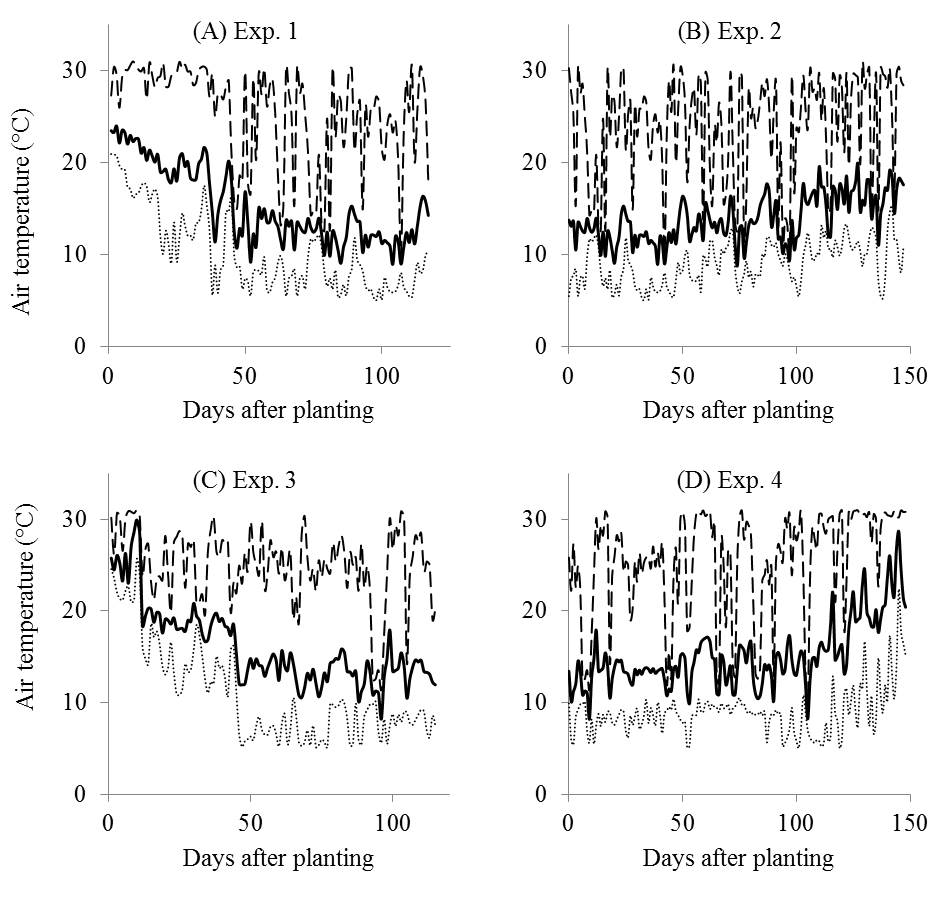

Supplement: Figure S1 — Daily mean, maximal and minimal air temperature at the height of 1.5 m above ground inside the greenhouse during (A) Exp. 1, (B) Exp. 2, (C) Exp. 3, and (D) Exp. 4. Solid curve is daily mean air temperature, dashed curve on top is the daily maximal air temperature, dotted curve at bottom is the daily minimal air temperature. [file Image1.jpeg]

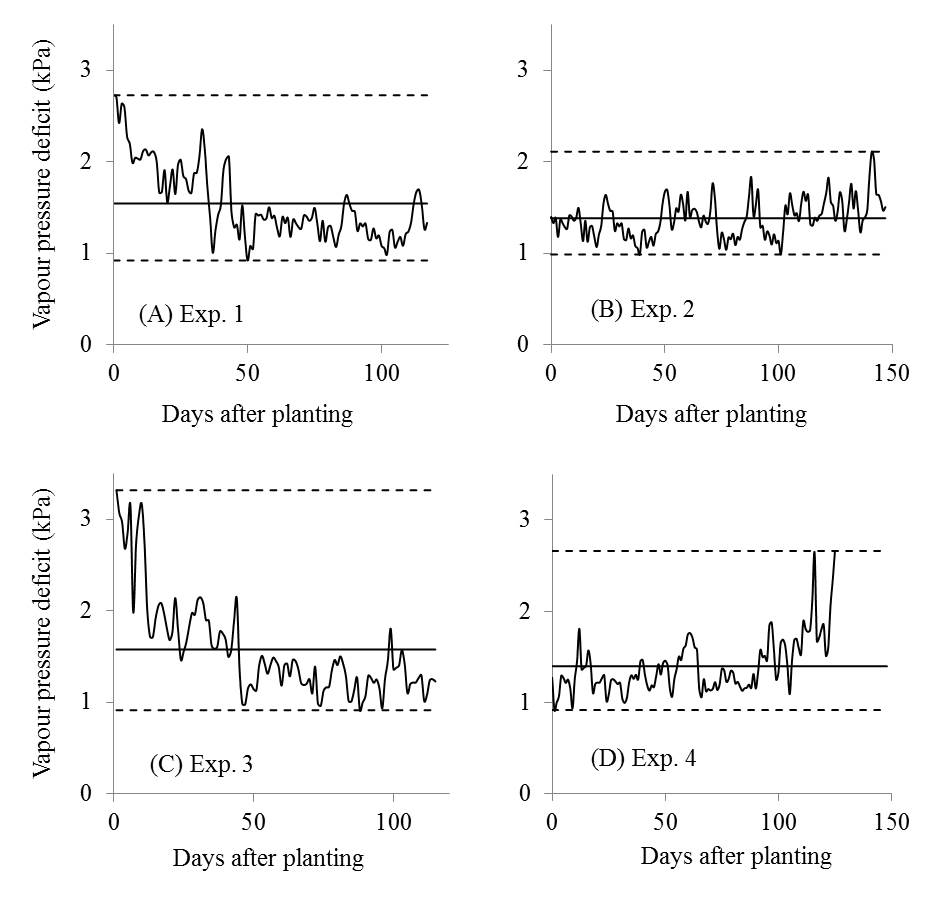

Supplement: Figure S2 — Vapor pressure deficit (VPD) at the height of 1.5 m above ground inside the greenhouse during (A) Exp. 1, (B) Exp. 2, (C) Exp. 3, and (D) Exp. 4. Curve is daily mean VPD, solid line is the average daily mean VPD during the whole growth period, and dashed lines are the maximal and minimal daily mean VPD during the whole growth period, respectively. [file Image2.jpeg]

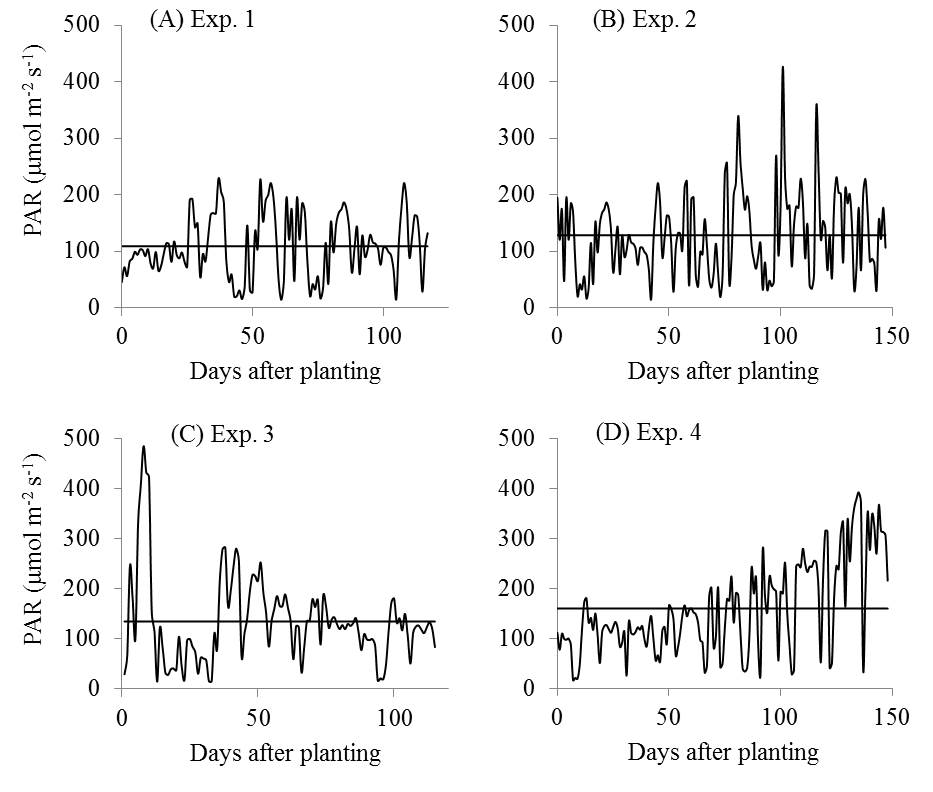

Supplement: Figure S3 — Daily mean photosynthetic active radiation (PAR) above crop canopy inside the greenhouse during (A) Exp. 1, (B) Exp. 2, (C) Exp. 3, and (D) Exp. 4. Curve is daily mean PAR, and line is the average daily mean PAR during the whole growth period. [file Image3.jpeg]

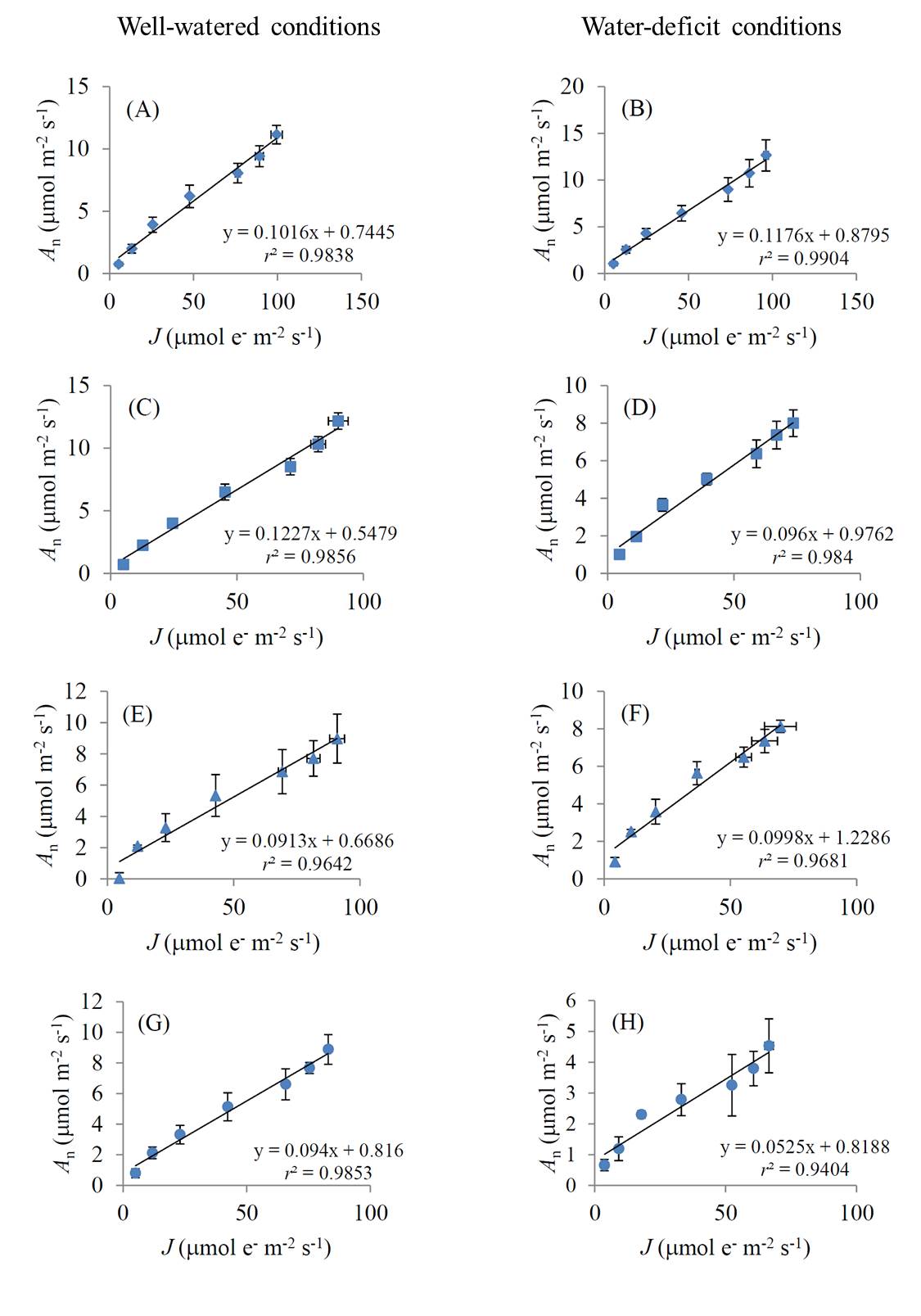

Supplement: Figure S4 — Relationships between An and J under well-watered conditions (A,C,E,G) and water-deficit conditions (B,D,F,H) (N85: A,B; N65: C,D; N45: E,F; N25: G,H). All data points were chosen from light levels at or below 1,000 μmol m−2 s−1 and leaf temperature at 20 ± 2°C (Vertical error bar indicates standard error of measured An; horizontal error bar indicates standard error of calculated J). [file Image4.jpeg]
